# Causal Inference for Heterogeneous Data and Information Theory

**DOI:** 10.3390/e25060910

**Published:** 2023-06-08

**Authors:** Kateřina Hlaváčková-Schindler

**Affiliations:** 1 Faculty of Computer Science, University of Vienna, 1090 Vienna, Austria; katerina.schindlerova@univie.ac.at; 2 Institute of Computer Science of the Czech Academy of Sciences, 182 00 Prague, Czech Republic

The present Special Issue of Entropy, entitled "Causal Inference for Heterogeneous Data and Information Theory", covers various aspects of causal inference. The issue presents thirteen original contributions that span various topics, namely the role of instrumental variables in causal inference, the estimation of average treatment effects and the temporal causal models. Four papers are devoted to the design of novel causal models using interventions. The contributions use approaches of information theory, probability, algebraic structures, neural networks and with them related machine learning tools. The papers range from the theoretical ones, the paper applying the models, to the papers providing software tools for causal inference. All papers were peer-reviewed and accepted for publication due to their highest quality contribution. Here, we shortly preview the topics of the contributions.

**Instrumental variable in causal inference.** Papers [1,2,3] investigate models using instrumental variable in causal inference. Paper [1] deals with the challenge to reconcile the approaches to causal inference based on independence of cause and mechanism, and approaches based on conditional independence. It is shown that methods based on the independence of cause and mechanism indirectly contain traces of the existence of hidden instrumental variables (IV). Paper [2] investigates the problem of selecting instrumental variables relative to a target causal influence X→Y from observational data generated by linear non-Gaussian acyclic causal models in the presence of unmeasured confounders. A necessary condition for detecting variables that cannot serve as instrumental variables is proposed. Paper [3] used the piecewise linear model to fit the relationship between the continuous instrumental variable and the continuous explanatory variable, as well as the relationship between the continuous explanatory variable and the outcome variable, which generalizes the traditional linear instrumental variable models.

**Estimating average treatment effect.** Papers [4,5,6,7] deal with the estimation of the Average Treatment Effect (ATE). Papers [4,5] approach the estimation of ATE using neural networks. The estimation of ATE as a causal parameter is carried out in two steps [4]. In the first step, the treatment and outcome are modeled to incorporate the potential confounders, and in the second step, the predictions are inserted into the ATE estimators such as the Augmented Inverse Probability Weighting (AIPW) estimator, based on neural networks (NN). Paper [4] proposed the normalization of AIPW (referred to as nAIPW) to overcome the drawbacks of AIPW. Paper [5] builds on [4] and uses architectures with an $L_{1}$-regularization on specific NN parameters and investigates how certain hyperparameters should be tuned in the presence of confounders and IVs to achieve a low bias-variance tradeoff for AIPW estimator.

Paper [6] contributes with the novel econometric software to the community dealing with causal inference and heterogeneous treatment effects estimation. The mcf package is an open-source Python package implementing Modified Causal Forest (MCF), a causal machine learner. For all resolutions of treatment effects estimation, which can be identified, the mcf package provides inference and novel insights on causal effect heterogeneity. The mcf constitutes a practical and extensive tool for a modern causal heterogeneous effects analysis.

Paper [7] investigates causal inference for heterogeneous treatment effects. The estimation of both overall and heterogeneous treatment effects can be hampered when data are structured within groups if one fails to correctly model the dependence between observations. Most machine learning (ML) methods do not readily accommodate such structures. Paper [7] introduces a new algorithm, stan4bart, that combines the flexibility of Bayesian Additive Regression Trees (BART) for fitting nonlinear response surfaces with the computational and statistical efficiencies of using Stan for the parametric components of the model. It is demonstrated how stan4bart can be used to estimate average, subgroup, and individual-level treatment effects with stronger performance than other flexible approaches.

**Temporal causal models.** Papers [8,9] consider causal models using time. Paper [8] investigates causal discovery in high-dimensional point process networks with hidden nodes. A big challenge in the multivariate causal discovery is the confounding problem. Paper [8] proposes a deconfounding procedure to estimate high-dimensional point process networks with only a subset of the nodes being observed. The method allows flexible connections between the observed and unobserved processes.

Paper [9] deals with the paradox of time in dynamic causal systems. It investigates the role of time in dynamic systems, where causes take continuous values and also continually influence their effects. A question is posed whether interacting with systems that unfold more slowly might reduce the systematic errors that result from these strategies. It is found that slowing the task indeed reduced the frequency of one type of error, albeit at the cost of increasing the overall error rate.

**Causal models and modeling under interventions.** Paper [10] examines the so-called interventional fairness with indirect knowledge of unobserved protected attributes. Often, the protected attribute is absent from the training dataset for legal reasons. However, datasets still contain proxy attributes that capture protected information and can inject unfairness in the ML model. Paper [10] examines systems flagging individual samples and considers a feedback-based framework where the protected attribute is unavailable and the flagged samples are indirect knowledge. The reported samples are used as guidance to identify the proxy attributes that are causally dependent on the (unknown) protected attribute. The work is done under the causal interventional fairness paradigm. Without requiring the underlying structural causal model a priori, an approach is proposed that performs conditional independence tests on observed data to identify such proxy attributes.

Paper [11] studies causal algebras on Chain Event Graphs (CEG). One popular causal analysis following Pearl [12] and Spirtes et al. [13] to study causal relationships embedded in a system is to use a Bayesian Network (BN). However, certain causal constructions that are particularly pertinent to the study of reliability are difficult to express fully through a BN. The previous work of the authors of [11] demonstrated that an event tree rather than a BN could provide an alternative framework that could capture most of the causal concepts needed within this domain. A causal calculus for a specific type of intervention, called a remedial intervention, was devised on this tree-like graph. Paper [11] builds on their previous work and shows that remedial maintenance interventions but as well as interventions associated with routine maintenance can be well-defined using this alternative class of graphical model.

Universal Causality is a mathematical framework introduced in [14]. This work is based on higher-order category theory, which generalizes previous approaches based on directed graphs and regular categories. The paper presents a hierarchical framework called Universal Causality Layered Architecture (UCLA), where at the top-most level, causal interventions are modeled as a higher-order category over simplicial sets and objects. Causal inference between layers is defined as a lifting problem, a commutative diagram whose objects are categories, and whose morphisms are functors that are characterized as different types of fibrations. UCLA is illustrated using a variety of representations, including causal relational models and other models.

Paper [15] develops a probabilistic theory of causation using measure-theoretical concepts and information-theoretic functionals and suggests practical routines for conducting causal inference. The theory is applicable to both linear and high-dimensional nonlinear models. It is shown that the suggested measure-theoretic approaches do not only lead to better predictive models, but also to more plausible parsimonious descriptions of possible causal flows.

We are convinced that this heterogeneous collection of outstanding papers on causal inference extends the knowledge of the community working in causal inference both in theory and practical applications. We wish the readers a lot of joy by reading.
